# Strategies for Implementing an Electronic Patient-Reported Outcomes-Based Symptom Management Program Across Six Cancer Centers

**DOI:** 10.21203/rs.3.rs-3879836/v1

**Published:** 2024-01-24

**Authors:** Michael J. Hassett, Samira Dias, Christine Cronin, Deborah Schrag, Nadine McCleary, Jaclyn Simpson, Tiana Poirier-Shelton, Jessica Bian, James Reich, Don Dizon, Megan Begnoche, Hannah Hazard Jenkins, Laura Tasker, Sandra Wong, Loretta Pearson, Roshan Paudel, Raymond U. Osarogiagbon

**Affiliations:** Dana-Farber Cancer Institute; Dana-Farber Cancer Institute; Dana-Farber Cancer Institute; Memorial Sloan Kettering Cancer Center; Dana-Farber Cancer Institute; Baptist Medical Center; Baptist Medical Center; MaineHealth; MaineHealth; Lifespan Cancer Institute and Brown University; Lifespan Cancer Institute and Brown University; West Virginia University Cancer Center; West Virginia University Cancer Center; Dartmouth-Hitchcock Medical Center; Dartmouth-Hitchcock Medical Center; Dana-Farber Cancer Institute; Baptist Medical Center

**Keywords:** Consolidated Framework for Implementation Research (CFIR), ePROs (electronic patient-reported outcomes), Symptom management, Expert Recommendations for Implementing Change (ERIC), Implementation strategy, Implementation science

## Abstract

**Background:**

Electronic patient-reported outcome (ePRO)-based symptom management improves cancer patients’ outcomes. However, implementation of ePROs is challenging, requiring technical resources for integration into clinical systems, substantial buy-in from clinicians and patients, novel workflows to support between-visit symptom management, and institutional investment.

**Methods:**

The SIMPRO Research Consortium developed eSyM, an electronic health record-integrated, ePRO-based symptom management program for medical oncology and surgery patients and deployed it at six cancer centers between August 2019 and April 2022 in a type II hybrid effectiveness-implementation cluster randomized stepped-wedge study. Sites documented implementation strategies monthly using REDCap, itemized them using the Expert Recommendations for Implementation Change (ERIC) list and mapped their target barriers using the Consolidated Framework for Implementation Research (CFIR) to inform eSyM program enhancement, facilitate inter-consortium knowledge sharing and guide future deployment efforts.

**Results:**

We documented 226 implementation strategies: 35 ‘foundational’ strategies were applied consortium-wide by the coordinating center and 191 other strategies were developed by individual sites. We consolidated these 191 site-developed strategies into 64 unique strategies (i.e., removed duplicates) and classified the remainder as either ‘universal’, consistently used by multiple sites (N=29), or ‘adaptive’, used only by individual sites (N=35). Universal strategies were perceived as having the highest impact; they addressed eSyM clinical preparation, training, engagement of patients/clinicians, and program evaluation. Across all documented SIMPRO strategies, 44 of the 73 ERIC strategies were addressed and all 5 CFIR barriers were addressed.

**Conclusion:**

Methodical collection of theory-based implementation strategies fostered the identification of universal, high-impact strategies that facilitated adoption of a novel care-delivery intervention by patients, clinicians, and institutions. Attention to the high-impact strategies identified in this project could support implementation of ePROs as a component of routine cancer care at other institutions.

## Background

Symptom control in cancer patients is often reactive and suboptimal (1). To address this gap, investigators have explored the collection and utilization of electronic patient-reported outcomes (ePROs) in the homecare setting (2). These explorations have demonstrated that proactive, routine collection of ePROs informs clinical care and improves symptom control in medical oncology and surgical practice (3). For example, ePRO-based symptom monitoring has been shown to decrease symptom burden (4), improve quality of life (5), reduce acute care utilization (6) and extend survival (2). Despite these favorable findings, ePRO use in routine clinical practice remains uncommon. Implementing ePROs is challenging, because it requires technical resources, robust multi-stakeholder support from patients, providers, institutional leaders, and technical support teams, and substantial institutional investment (7). While a few studies have demonstrated that ePRO-based programs can be deployed successfully, there is a paucity of data defining the implementation strategies that are most likely to lead to program success (8–10). Lack of knowledge regarding best practices for effective implementation confounds efforts to expand these programs to new institutions.

The **S**ymptom Management **Im**plementation of **P**atient-**R**eported **O**utcomes in Oncology (SIMPRO) Research Center was formed in 2019 to develop and deploy a novel ePRO-based Symptom Management (eSyM) program across six diverse health systems. To foster program adoption and maintenance, eSyM was fully integrated into the electronic health record and patient portal system produced by Epic (Verona, WI). The eSyM program, which includes symptom questionnaires, patient tip sheets, decision support alerts, clinical reports, and documentation aides, was designed to facilitate symptom management for patients starting chemotherapy or recovering from surgery in the routine cancer care setting (7). In the context of a type II hybrid implementation-effectiveness study, eSyM was deployed by six institutions in a staggered fashion from 2019 to 2022. Throughout this project, we sought to identify and systematically characterize implementation strategies across SIMPRO institutions to, 1) optimize ePRO clinical implementation, 2) identify high-impact strategies that could facilitate similar implementation efforts at other institutions, and 3) characterize a prospective approach to implementation strategy identification and characterization.

Considering that eSyM was deployed across multiple sites, each with its own structure and culture, robust conceptual frameworks were needed to classify and categorize the implementation strategies. The Expert Recommendations for Implementing Change (ERIC) (11, 12) compiled a refined set of terms and definitions to improve the clarity, relevance, and comprehensiveness of efforts to classify implementation strategies. And the Consolidated Framework for Implementation Research (CFIR) (13) contextualized the settings in which barriers to implementation exist. CFIR groups barriers into five domains – innovation, outer setting, inner setting, individuals, and implementation process – and provides a practical theory-based guide for assessing potential barriers and facilitators to guide tailoring of implementation strategies (11, 13). Using a matching tool, ERIC strategies can be linked to the CFIR barriers they address (14). The ERIC and CFIR frameworks have been used retrospectively to characterize the implementation of various healthcare delivery interventions (15, 16), though they have not been frequently used to characterize the implementation of ePRO-based symptom management programs. We decided to use CFIR and ERIC prospectively to document implementation barriers and facilitators and to systematical categorized implementation strategies during the implementations of eSyM. These frameworks provided a consistent and generalizable structure to support comparative analysis across SIMPRO’s institutions and to foster development of findings that generalize beyond the consortium.

## Methods

### SIMPRO Research Consortium

The SIMPRO Research Consortium includes six US-based cancer centers: Dana-Farber Cancer Institute (Boston, MA), Lifespan Cancer Institute (Providence, RI), Maine Medical Center (Portland, ME), West Virginia University Cancer Institute (Morgantown, WV), Dartmouth Hitchcock Medical Center (Lebanon, NH), and Baptist Memorial Health Care Corporation (Memphis, TN) (7, 17). SIMPRO is one of three research centers in the National Cancer Institute’s Improving the **M**anagement of sym**P**toms during **A**nd following **C**ancer **T**reatment (IMPACT) Cancer Moonshot Consortium. IMPACT was designed to study the pragmatic implementation of ePROs for symptom control across the cancer care continuum including curative-intent treatment, palliative care, and survivorship care (18). SIMPRO developed and deployed the eSyM electronic symptom management program so that it was fully integrated into the Epic electronic health record (7). The program enables patients who recently started chemotherapy or had surgery to report symptoms through a secure patient portal; and it allows clinicians to track and respond to patient-reported symptoms between clinic visits. SIMPRO sites implemented eSyM for routine medical oncology and surgical care among patients with gastrointestinal, thoracic, and gynecologic conditions (17). The Western Institutional Review Board, which approved the protocol as a minimal risk study, waived the need for individual authorization for release of health data and informed consent.

### Implementation Plan and Data Collection

**Figure 1:**
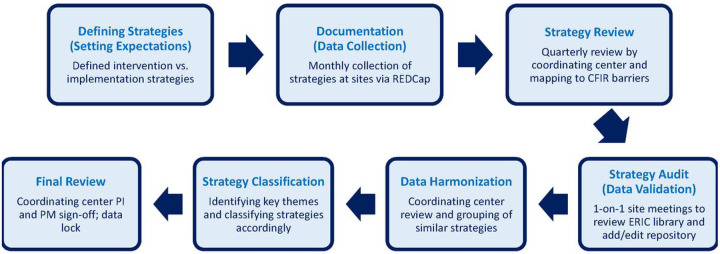
Overview of SIMPRO Strategy Documentation, Audit, and Analysis Process. This process was designed to inform eSyM program enhancements, facilitate inter-consortium knowledge sharing, and guide future deployment efforts.

Implementation included planning, development, deployment, and optimization. Each site provided its own implementation team, including a principal investigator, project manager, medical informatics technical lead, clinical champions, operations stakeholders, and patient representatives. Dana-Farber Cancer Institute supported all site implementation efforts as the coordinating center. We used a systematic approach to document, classify, review, harmonize, and analyze implementation strategies (Fig. 1). eSyM development and pilot testing began in the Fall of 2018. Informal strategy documentation started at this time, with formal strategy documentation occurring from April 2020 through March 2022. During the formal strategy documentation period, ongoing or critical strategies that were implemented prior to April 2020 were added to the documentation repository. The coordinating center created a common REDCap-based tracking tool that each site used to document its implementation strategies.

**Figure 2:**
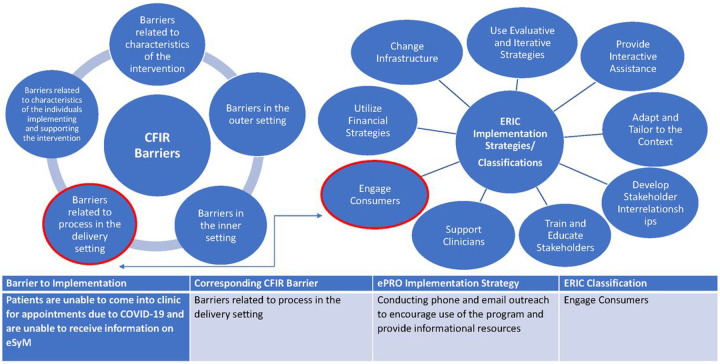
Example of SIMPRO Strategies Mapped Using the CFIR-ERIC Frameworks. Together, the CFIR and ERIC frameworks form a matching tool linking CFIR barriers with corresponding ERIC implementation strategies.^19^ This allowed us to identify all the SIMPRO implementation strategies used to address each implementation barrier. The figure shows an example of how this mapping was applied to one implementation strategy.

Site project managers updated the REDCap strategy tracker monthly when they recorded new implementation efforts and revised previously documented strategies. All site project managers underwent training prior to the initiation of strategy documentation to review of the REDCap documentation system, descriptions of how and when to use it, and key definitions for classifying strategies in a consistent and cohesive manner. Data elements collected by the tracker included a brief description, dates of usage, the intended audience, and other features (**Supplemental Table 1**). Site project managers classified each documented strategy using the ERIC framework and identified the barriers addressed by the strategy using the CFIR framework (Fig. 2). We considered ERIC the primary classification framework and CFIR as secondary. The coordinating center was responsible for documenting implementation strategies used to promote the program consortium-wide.

### Strategy Review

To ensure uniformity in documentation and classification, the coordinating center conducted quarterly reviews of the strategy repository, monitored site progress in documentation, identified inconsistencies, confirmed ERIC strategy classifications, and assessed the mapping of implementation strategies to the CFIR framework. When the initial review found incomplete documentation and inconsistent strategy classification, we created definitions to distinguish between ‘implementation strategy’ and the intervention. We also created a data dictionary to improve consistency in the categorization of strategies. For example, references to “consumers” were interpreted as referencing patients and “stakeholders” were clinical and research staff. After strategy documentation was complete, the coordinating center, site project managers, and site principal investigators harmonized all strategies in the repository. This was done to remove strategies that were classified as part of the intervention rather than the implementation; to modify previously documented strategy categorizations to ensure consistency across sites (e.g., if a site documented separate strategies for patient outreach via phone when conducted by distinct staff at different times, these were consolidated into one phone outreach strategy); to modify classifications to ensure consistent use of the CFIR and ERIC frameworks; and to add missing strategies.

### Strategy Analysis

Analysis focused on three areas: describing key characteristics, such as duration of use and reason for discontinuation; characterizing strategies relative to the ERIC and CFIR frameworks; and identifying thematic patterns across strategies. We developed qualitative codes to aid classification and mapped the strategies to these codes: 1) *foundational strategies* were orchestrated by the coordinating center and systematically deployed across the consortium; 2) *universal strategies* were managed by each site but used commonly across multiple sites; and 3) *adaptive strategies* were used exclusively by one site to address an institution-specific barrier. Two independent reviewers classified the strategies and identified thematic patterns; a third reviewer adjudicated discrepancies. All documented strategies were maintained in the REDCap repository for reference. We assessed the number of strategies that were inactivated during the study and collected reasons for inactivation. All analytic work was conducted using Microsoft Excel. The SIMPRO implementation strategy repository was locked for analysis on March 31, 2022. The coordinating center program manager and principal investigator approved the data set for analysis on April 29, 2022.

## Results

**Figure 3:**
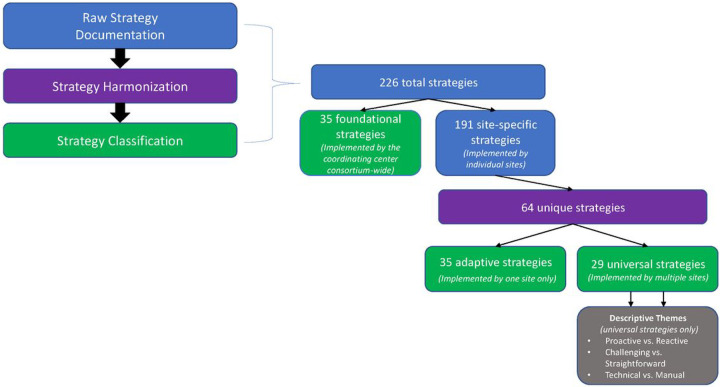
Summary of Implementation Strategy Documentation, Harmonization, and Classi cation. The study identified 109 unique strategies: 35 foundational (implemented by the coordinating center), 35 adaptive (implemented by one site), and 29 universal (implemented by all sites)

In total, 226 distinct implementation strategies were documented – 191 by individual sites and 35 by the coordinating center (Fig. 3). We categorized all 35 consortium-wide strategies as foundational, because they were critical to the development and deployment of the system. The 191 strategies documented by the sites contained duplicates because different sites frequently documented similar strategies. After de-duplication, there 64 unique site-specific strategies. Of these, 29 were categorized as universal strategies that were used across multiple sites, and the remaining 35 were categorized as adaptive strategies that were used only at one site. ERIC and CFIR mappings were assessed for all unique strategies. Of the 73 possible ERIC strategies, 44 (60%) were documented at least once and 29 were not employed by any site (Table 1). All 5 CFIR barriers were targeted.

### Foundational strategies

Among the 35 foundational strategies, the most common ERIC category was: *use evaluative and iterative strategies*. Examples included collecting patient and clinical evaluation surveys before and after program deployment; conducting preparatory site visits before program deployment; identifying technical leads, project managers, and data analysts; and holding monthly calls with staff from all six sites to discuss progress. Foundational strategies were initiated at different times throughout the project: 51% before, 14% during, and 24% after go-lives. Most strategies took less than an hour to complete and involved onetime actions (e.g., executing data use agreements, identifying data experts, introducing technical enhancements). The CFIR barrier most frequently address by the foundations strategies *was related to process in the delivery setting* (N = 13) and the least frequently addressed was *related to the inner setting* (N = 3). Although strategies could address more than one CFIR barrier, most implemented strategies targeted only one barrier. A full list of the foundational strategies and their ERIC and CFIR classifications can be found in **Supplemental Table 2.**

### Universal strategies

Each universal strategy was used by more than one site, highlighting that many strategies were repeated across SIMPRO sites. Among the 29 universal strategies, the most common ERIC categories were: 1) *use evaluative and iterative strategies* to address real-time barriers and assess intervention progress, *2) train and educate stakeholders* to prepare key stakeholders and incorporate the intervention into their daily work, *and 3) engage consumers* to inform, educate and encourage end users to interact with a new program. Examples of specific strategies for all these categories can be found in **Supplemental Table 3.** Fifty-two percent were implemented after go-live, 31% occurred before go-live, and 17% started during go-live. The time to deploy strategies was variable. For example, making patient phone calls took 3–5 minutes, whereas expanding the eSyM program to new clinics took as long as a week. Most strategies were used once (48%). Most were implemented by study staff exclusively (66%), with only 3% being deployed by a site’s clinic or administrative staff. The most frequently addressed CFIR barrier was *related to process in the delivery setting* (N = 20) and the least frequently addressed CFIR barrier was related to *the outer setting* (N = 1). Strategies also addressed *barriers related to characteristics of the individuals implementing the intervention* (N = 12), *characteristics of the intervention* (N = 5), and *barriers in the inner setting* (N = 5).

**Figure 4:**
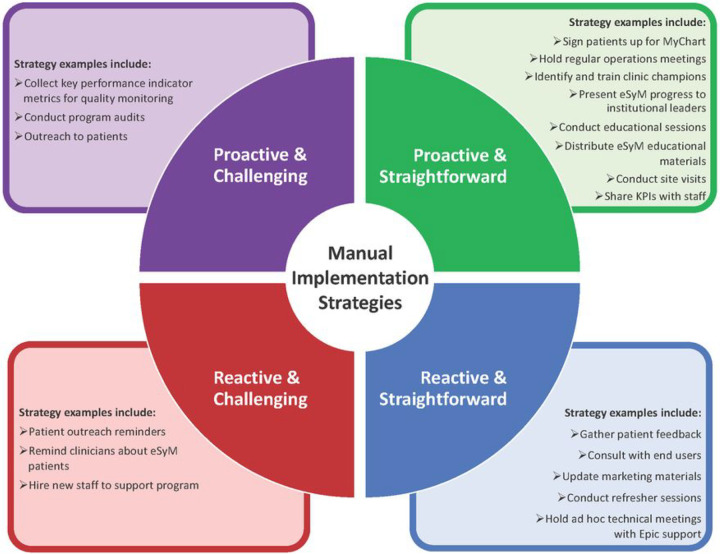
Summary of Thematic Sub-Classi cations for Identi ed Universal (High Impact) Strategies. Focusing on the 29 strategies that were implemented by all sites, and therefore considered universal, each was characterized as either proactive or reactive and as either straightforward or challenging, to inform deployment strategies at other organizations.

Since universal strategies were organically implemented across multiple sites, we concluded that they were likely important, generalizable, and of interest to other institutions. So, we conducted qualitative analyses to characterize the similarities and differences between these strategies across SIMPRO sites. This led to the identification of three thematic patterns: 1) straightforward versus challenging; 2) technical versus manual; and 3) proactive versus reactive. Straightforward strategies were easy to integrate into the workflow whereas challenging strategies required extensive resources. Technical strategies required use of or integration with the EHR, whereas manual strategies only required staff involvement. Proactive strategies were typically planned before implementation, whereas reactive strategies were developed in response to problems that were identified after ‘go-live’. We identified 20 straightforward versus 9 challenging, 4 technical versus 25 manual, and 17 proactive versus 12 reactive strategies (Fig. 4, **Supplemental Table 4**).

### Adaptive strategies

Adaptive strategies were developed to meet site-specific implementation challenges. Among the 35 adaptive strategies, the most common ERIC categories were *use evaluative and iterative strategies* and *develop stakeholder interrelationships* (9 strategies each). Examples of adaptive strategies included: changing the build of the ePRO program in the EHR to accommodate a site-specific need, changing the outreach strategy to address issues with patient engagement, or changing the clinical workflow to integrate the ePRO program into day-to-day practice and increase clinician engagement. Adaptive strategies allowed the implementation team to address regional, clinical, and provider differences in cancer care. Eighty percent of adaptive strategies were reactive to unforeseen site-specific implementation challenges, with only 14% occurring before and 6% during go-live. Most strategies took less than an hour to implement (range: 1min – 10 hours). The CFIR barriers addressed in order of frequency were related to *process in the delivery setting* (N = 12), *characteristics of the individuals implementing the intervention* (N = 9), the *inner setting* (N = 7), *characteristics of the intervention* (N = 6), and *the outer setting* (N = 1). A full list of adaptive strategies can be found in **Supplemental Table 5.**

### Inactivated strategies

**Figure 5:**
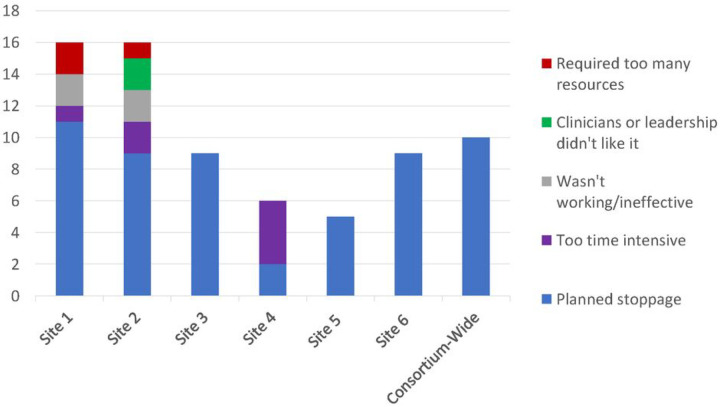
Implementation Strategies Inactivation Reasons. For the strategies that were inactivated, five reasons were identified.

Seventy-one of the originally documented 226 (31%) strategies were inactivated during the study: 10 foundational, 49 universal, and 12 adaptive (**Supplemental Table 6**). Reasons for inactivation included: planned stoppage (the strategy was meant to be time-limited), too resource intensive, ineffective, or inadequate stakeholder engagement (Fig. 5). Planned stoppage was the most common reason for inactivation (77%). Strategies inactivated for being “too time or resource intensive” (14%) often involved in-person patient outreach or regular meetings with program stakeholders/champions. Strategies inactivated because of ineffectiveness (6%) were usually related to patient outreach. Finally, strategies inactivated due to low stakeholder engagement (3%) involved the review of clinician schedules for eSyM eligible patients.

## Discussion

Few prior studies have described the strategies needed to support the deployment of ePRO-based symptom management programs. Consequently, the optimal methods for implementation remain uncertain. In the context of a prospective, hybrid effectiveness-implementation study, we prospectively and systematically documented and categorized the implementation strategies used by six cancer centers during their deployment of eSyM – an ePRO-based, EHR-integrated symptom management program. We found that all institutions used multiple implementation strategies (range: 25–41, mean: 32 per site) and that most strategies required support from dedicated project staff (i.e., resources beyond those typically available to sites). Taking a prospective and systematic approach to strategy documentation and evaluation facilitated intra-consortium collaboration and strategy adaptation which increased the likelihood of successful implementation.

The most frequently used, and likely most impactful strategies focused on strengthening clinician and patient engagement. Typically, this involved educating and encouraging patients (by telephone call, email, or patient portal), providing consistent user training, and distributing educational materials for patients and clinicians. Most strategies were used across all sites. This included universal strategies, which were managed independently by the sites, and foundational strategies, which were managed centrally by the coordinating center. The latter strategies alleviated some of the implementation burden placed on sites, helped ensure program consistency, and fostered program success. All sites employed adaptive strategies to address site-specific implementation challenges. It was not surprising that institutions needed to incorporate adaptive strategies, because they had to account for their sites’ unique structures, cultures, processes, policies, and staffing challenges. Many sites implemented strategies reactively – in response to needs that only became known after the initial program launched. For example, when clinician engagement waned, the team instituted clinician reengagement meetings and generated automated key performance indicator reports to monitor site progress. Altogether, these findings underscore the importance of including both standard and adaptive approaches to implementation to maximize the chance of success.

Three cross-cutting factors substantially influenced the effectiveness of implementation strategies: the bandwidth of existing staff, the availability of funding to hire dedicated resources, and institutional buy-in. The time of existing staff, especially those with knowledge of technical systems and clinical workflows, was needed to ensure the integration of eSyM into the EHR and its incorporation into clinical processes. Grant funding supported the hiring of a small number of project-specific staff, who facilitated each site’s use of critical implementation strategies and were essential for engaging patients and educating clinicians. Institutional buy-in ensured that existing staff could prioritize eSyM-related work and enabled hiring of dedicated resources. It was important, because the funding provided by the grant was insufficient to cover all the time and effort needed to deploy the program. In addition to supporting implementation, these three factors will be essential for ensuring the long-term sustainability of this program.

The most frequently employed ERIC strategy was, *use evaluative and iterative strategies*. Implementation strategies addressed all five CFIR barriers, with the barriers related to *process in the delivery setting* being targeted substantially more than the others. Prior studies that used the CFIR-ERIC frameworks to identify implementation barriers usually focused on the pre-implementation phase and addressed the provider and organizational levels (15, 19). We were able to analyze barriers throughout all phases of the project (i.e., pre-, peri-, and post-implementation); and to consider multiple levels, including those impacting the patient, clinician, and health-system.

### Limitations

The process used to identify strategies may not have captured all implementation efforts. Documenting and characterizing strategies across six centers over two years was time- and resource-intensive. Sites often documented similar strategies differently, which had to be re-classified for consistency. The processes used to characterize strategies and map them to CFIR and ERIC were somewhat subjective. We used a consensus-based approach to resolve differences, but still found that some strategies could be classified in multiple ways. This limitation derives partly from the non-specific nature of the conceptual frameworks. Efforts to evolve and improve these frameworks may be warranted. Some of the high-impact strategies that we identified may be specific to eSyM deployment in the SIMPRO consortium and may not be generalizable to other programs at other sites. The COVID pandemic had a major impact on the strategies that were used and the process for identifying strategies. We did not explore the associations between implementation strategies and quantitative measures of program utilization. We collected several key performance indicators (KPIs), and in a future study plan to analyze the relationships between specific strategies and these KPIs to validate our findings. Lastly, despite our efforts to support implementation robustly, we still found that maintaining patient engagement and staff buy-in was challenging.

## Conclusions

We described a rigorous approach to identifying and characterizing the high-impact strategies needed to address major barriers to the implementation of an ePRO-based symptom management program. Deploying a complex program across six different sites meant that multiple strategies were needed to address a variety of barriers. Foundational strategies ensured efficiency and consistency across sites, universal strategies offered high-impact solutions for diverse settings, and adaptive strategies addressed site-specific barriers. The most impactful strategies fostered the engagement of patients, clinicians, and operations staff through regular outreach and education. Many strategies were time- and resource-intensive, sometimes leading to their inactivation due to resource constraints.

Our findings emphasize the inherent need for a multifaceted and multi-modal approach that allocates clinical and operational staff, addresses technologic and human-based challenges, and ensures the availability of EHR-system resources and funding. Acknowledging that resource constraints limit the number of implementation strategies any one site can deploy, having a central team oversee a set of high-impact universal strategies increased efficiency and facilitated program deployment. Conducting a prospective, systematic evaluation of implementation strategies concurrent with program deployment allowed sites to learn from each other, thereby increasing the effectiveness and sustainability of the intervention. The relative success of this approach underscores the importance of incorporating prospective identification and characterization of implementation strategies as a component of agile project management to foster real-time learning.

Undeniably, successful implementation of ePROs into routine care requires significant technical resources, substantial buy-in from patients and clinicians, institutional investment, and deployment of novel workflows to support between-visit symptom management. Other health systems looking to implement ePRO-based programs, especially those with limited resources, should, 1) focus on deploying EHR-embedded versus free-standing programs; 2) prioritize use of the high-impact universal implementation strategies identified by our analysis; 3) be prepared to introduce adaptive strategies to address dynamic challenges; and 4) look for partnerships and collaborations to support and centrally coordinate efforts thereby reducing the burden on individual Institutions.

## Figures and Tables

**Table 1:** List of ERIC Strategies Documented Throughout the SIMPRO Consortium.

Strategy Classification	Strategy Sub-classification	Count Across SIMPRO
Use Evaluative and Iterative Strategies	Assess for readiness and identify barriers and facilitators	5
	Audit and provide feedback	6
	Conduct cyclical small tests of change (e.g., PDSA cycles)	2
	Conduct local needs assessment	2
	Develop a formal implementation blueprint	2
	Stage implementation scale up	5
	Develop and implement tools for quality monitoring	14
	Obtain and use patients/consumers and family feedback	7
	Purposefully reexamine the implementation	9
Provide Interactive Assistance	Facilitation	3
	Provide local technical assistance	3
Adapt and Tailor to the Context	Promote adaptability of the EBP	6
	Tailor strategies	4
	Use data experts	3
	Use data warehousing techniques	1
Develop Stakeholder Interrelationships	Build a coalition	1
	Capture and share local knowledge	7
	Involve executive boards	3
	Obtain formal commitments	1
	Promote network weaving	1
	Identify early adopters	4
	Inform local opinion leaders	1
	Identify and prepare champions	7
	Organize clinician implementation team meetings	2
	Visit other sites	2
Train and Educate Stakeholders	Conduct educational meetings	11
	Conduct educational outreach visits	3
	Develop educational materials	7
	Shadow other experts	1
	Conduct ongoing training	7
	Create a learning collaborative	1
	Distribute educational materials	5
	Provide ongoing consultation	7
	Use train-the-trainer strategies	6
Support Clinicians	Create new clinical teams	2
	Facilitate relay of clinical data to providers	8
	Remind clinicians	8
Engage Consumers	Intervene with patients/consumers to enhance uptake and adherence	23
	Involve patients/consumers and family members	2
	Prepare patients/consumers to be active participants	16
	Use mass media	6
Utilize Financial Strategies	Access new funding	1
	Alter incentive/allowance structures	1
Change Infrastructure	Change physical structure and equipment	2
	Other: ____________	8
**TOTAL**		**226**

This table includes a raw count of all documented strategies for each ERIC subclassification prior to strategy harmonization. Of the 73 available ERIC strategies, 44 were documented at least once during program implementation and 29 were not employed at any of the SIMPRO sites.

## Data Availability

The datasets used and/or analyzed during the current study are available from the corresponding author on reasonable request.

## Abbreviations

ePROs: Electronic Patient-Reported Outcomes
SIMPRO: Symptom Management Implementation of Patient-Reported Outcomes in Oncology
IMPACT: Improving the Management of Symptoms During and Following Cancer Treatment
CFIR: Consolidated Framework for Implementation Research
eSyM: Electronic Symptom Management
ERIC: Expert Recommendations for Implementing Change
EHR: Electronic Health Record
